# DE Chronic diseases

**DOI:** 10.1093/eurpub/ckac131.127

**Published:** 2022-10-25

## Abstract

This abstract has been withdrawn

